# Long-term outcome after renal transplantation in childhood

**DOI:** 10.1007/s00467-007-0559-2

**Published:** 2009-03-01

**Authors:** Lesley Rees

**Affiliations:** 1 Department of Nephrology, Great Ormond Street Hospital for Children NHS Trust, London, UK; 2 Department of Nephrourology, Great Ormond Street Hospital for Children NHS Trust, Great Ormond Street, London, WC1N 3JH UK

**Keywords:** Long-term outcome, Mortality, Graft survival, Growth, Psycho-social

## Abstract

The purpose of this article is to review:

1. Factors influencing long-term outcome data after transplantation
2. Patient survival overall, the effect of recipient age and donor type, causes of death, comparison of mortality after transplantation with that on dialysis, and effect of pre-emptive transplantation and race
3. Transplant survival overall, and the effect of recipient and donor age, donor type, pre-emptive transplantation, recurrent diseases, human leukocyte antigen (HLA) matching, immunosuppression, concordance, hypertension, bladder dynamics and type of donor nephrectomy
4. Final height and obesity
5. Psycho-social outcome

## Introduction

The first paediatric renal transplantations took place in the 1970s. During the 1980s there was a steady increase in numbers, and there has been relative stabilisation since 2000, such that now most registries report that approximately two-thirds of children and adolescents on end-stage renal failure (ESRF) programmes have a transplant [1–4]. Studies of outcome over 20 years are now beginning to emerge [1, 5–9]. Such information is of considerable importance in order for us to counsel patients and families. However, we have to remember that we cannot assume to be able to extrapolate directly from these data to the current day. This is because changes over the years have had both positive and negative influences: advances in technical and therapeutic knowledge and the increasing numbers of families that are coming forward for living donation (LD) have to be balanced against the acceptance of more and more challenging patients onto ESRF programmes, including neonates and children with severe, and sometimes life threatening, co-morbidity [10]. What is consistently demonstrated is that there has been an improvement in outcome over the years [1, 11–13]. Clearly, it is rather artificial for us to consider long-term outcome only in patients with transplants, as most will switch treatment modalities over such a time span. Still, such data are important in order to provide comparative results between dialysis and transplantation, information which will influence management decisions.

### Factors influencing outcome data

Long-term outcome data will be affected by several factors. Available resources [7, 14] affect patient selection, follow-up and drug regimens. Community attitudes influence decisions regarding treatment of high-risk patients such as neonates and children with handicaps [15]. Some diagnoses are more common in particular ethnic backgrounds [2] or even countries (e.g. Finnish-type nephrotic syndrome), and patient and graft survival are affected by racial group [11].

Figures will also be affected by the proportion of LD, for which outcome is generally superior. Countries vary in the size of their LD programmes. Some centres use only LDs [16], whereas percentages differ considerably between both European countries and the USA. What is clear is that an increasing number of families are opting for LD, presumably as a result of information showing its superior results, at least over the first 5 years, the increasing understanding of the complications of dialysis, and the ability to plan the timing of the transplantation. For example, LDs provided 43% of all transplants in the USA between 1987 and 1991, 52% between 1987 and 2004, and, since 1998, have accounted for 58%. Of these donors, 82% were parents (56% mothers, 44% fathers) [17].

### Patient survival

The number of children on the waiting list for deceased donor (DD) transplantation has increased slowly over the past decade, despite an increasing proportion of LD. The mortality rate of children on the waiting list over the same time period has been low and has declined to 55 per 1000 at risk [18]. However, the overall mortality rate remains high, with a relative risk of death after transplantation that is 12.7-times higher than that of the age-related general population [1], with little sign of improvement since the 1980s [4, 19]. Although these values may be due to changing patient selection, they remain unacceptably high.

#### Overall patient survival

Overall 5-year patient survival varies between 70% and 100% at 5 years [1, 5–7, 9, 12, 13, 16, 18, 20–26], 75% to 95% at 10 years [1, 7, 9, 12, 18, 21, 23–26], 83% to 94% at 15 years [1, 27], 54% to 86% at 20 years [1, 5, 7] and there is one report of 81% 25-year patient survival [6].

#### Effect of recipient age

Age is an important factor that has been demonstrated to affect patient survival, although there is evidence that its effect has declined in recent years. Several early reviews showed increased mortality in young children and, particularly, in those under 2 years of age at transplantation: in two studies there was a mortality rate of 21% [5, 28]; however, more recently, survival rates of over 98% have been reported [29, 30]. Increased risk of death was also seen to extend up to age 5 years at transplantation: Dutch data showed that the mortality rate was nearly twice as high in those under 5 years of age than in those 6 to 10 years old [19]; in a review of children with an average age of 4.7 years who weighed <20 kg at transplantation, 19% were reported to have died [23]. A reduction in mortality rate has occurred in this age range too, with a halving of the mortality rate between the 1970s and 1980s in the Dutch report [19], a mortality rate of 8.2% unaffected by age less than or older than 5 years at transplantation [1], a 5-year survival rate of over 97% in under 6 year olds [31], a 91% 10-year survival rate in those weighing <15 kg [25], and 87% 15-year survival in those <11 kg at transplantation [32]. Most studies have small numbers of subjects, making statistics and interpretation difficult; however, what does seem to be the case is that deaths in those who had received transplants before they were 5 years old occur sooner after transplantation (indeed all the deaths in the younger age group were in those less than 15 years of age) than in those that had received transplants when they were over this age [1].

#### Effect of donor type

All reports show a small but consistent benefit of LD on mortality at all ages up to 5 years after transplantation [1, 12, 13, 18]. However, thereafter, there are few long-term data available, with one centre showing a benefit [12] and one not [1] at 10 years after transplantation. The survival rate for recipients of LDs has been improving over time: in the USA it was 96.1% 5 years after transplantation, between 1995 and 2004, and was 94.7% between 1987 and 1994; for infants, 3-year survival rates have improved from 88.4% between 1987 and 1994 to 94.9% since 1995. LD has a particular benefit for the very young child: 5-year patient survival rates for recipients less than 2 years old was 86% following living related donation (LRD) and 70% following DD transplantation [17].

#### Causes of death after transplantation

The major causes of death after transplantation are cardiovascular disease (CVD), infection and malignancy, variously reported as 30–36% for CVD, 24–56% for infection and 11–20% for malignancy [19, 21, 22, 27]. CVD has been defined in different ways, some studies including cerebrovascular events and arrhythmias as part of the definition. However, despite this, results are remarkably similar between centres, and, overall, CVD is the most common, and potentially preventable, cause of death. Infection, both sepsis related and due to opportunistic organisms, is becoming more of a problem with the use of more powerful immunosuppression. Malignancy is ten-times more common than expected for age [33, 34] and also might be expected to increase in incidence with current use of increasingly potent immunosuppression. Skin cancer is the most frequent, accounting for approximately 60% of all cancers, but it does not contribute to mortality. Non-Hodgkin’s lymphoma represents about a quarter of cases and is the commonest cancer to cause death. Most patients do not present until they have been moved to adult units; by 25 years after first renal replacement therapy (RRT), the probability of developing a malignancy is 17%, with a peak incidence at 15 years [34]. In some children risk may be heightened by syndromes associated with a genetic predisposition to cancer. The overall mortality rate, if Epstein–Barr virus (EBV)-driven post-transplantation lymphoproliferative disease (PTLD) is included, is 20%, and is associated with a 20% risk of graft failure [35].

Two other important factors that contribute to death are non-concordance with medications, or treatment withdrawal [1], and obesity [36]. Obese children aged 6 to 12 years had a higher risk of death than non-obese patients (adjusted relative risk 3.65 for LD; 2.94 for DD), and death was more likely to be as a result of cardiopulmonary disease (27% in obese children, 17% in non-obese children).

#### Comparison of mortality after transplantation with mortality on dialysis

All studies show a survival advantage for patients who receive transplants in comparison with those who undergo dialysis, be that peritoneal (PD) or haemodialysis (HD): the lifespan of a child on dialysis is 40–60 years less, and, for a child with a transplant, 20–25 years less, than that of age- and race-matched general populations [37]. Eighty percent of patients on HD, 83% of those on PD and 93% of those with a transplant survive 5 years, according to the latest (2006) United States Renal Data System (USRDS) data [3]. Mortality rates are seven-times higher in those who have been on dialysis for longer than they have had a transplant [38], and treatment with dialysis is associated with a risk more than four-times as high as for renal transplantation [4].

Most of the increased mortality in children on dialysis is due to CVD; indeed, prolonged stay on dialysis is the most important risk factor for CVD. USRDS data from 1990 to 1996 among patients who started ESRF therapy as children and had died before they were 30 years of age showed that transplant recipients had a 78% lower risk of cardiac death than did dialysis patients. The cardiac death rate among dialysis patients was approximately 21 per 1,000 patient-years, whereas after transplantation, values were under 2 per 1,000 patient-years [39]. CVD accounts for 57% of deaths of those on HD, 43% of those on PD and 30% of those with a transplant [4].

It might be expected, therefore, that patient survival after pre-emptive transplantation would be superior, although, conceivably, this might be difficult to demonstrate, as, for many children, the period of dialysis is only short. Eurotransplant identified a superior survival at 6 years in patients who received pre-emptive LD kidneys in comparison to LD after dialysis, but no difference in those receiving DD kidneys [40]. The North American Pediatric Renal Transplant Cooperative Study (NAPRTCS) showed that adolescents who had undergone pre-emptive transplantation had a better survival rate [41].

#### Effect of race

US registries demonstrate poorer outcomes for Afro-Americans than for the white population. Most of this difference can be accounted for by an increased incidence of cardiovascular deaths by approximately 1.6 times. Between 1990 and 1996, of patients who started RRT as children and had died before they were 30 years of age, the percentage of cardiac deaths was higher among black patients at all ages: 0–4 years, 36%; 5–9 years, 18%; 10–14 years, 35%; 15–19 years, 22%; 20–30 years, 32%; white patients 18%, 12%, 17%, 14%, and 23%, respectively. Among black patients, cardiac deaths occurred in 11% of transplant recipients and 34% of dialysis patients, and, among white patients, 9% and 25%, respectively [3, 11, 39, 42].

### Transplant survival

#### Overall transplant survival

Transplant survival has shown a steady increase over the years [1, 12, 16, 42]. The earliest transplants, prior to 1983, had only a 20% 10-year survival [1]. Over the next decade, following the introduction of ciclosporin, there was an improvement to 45% [1], and this percentage has continued to increase since, to as high as 95% at 10 years in one centre [12]. However, we are still seeing the effects of the early poor success rates: 25% of the early transplants failed at 5 years, yielding a projected half-life of 10 years. Given a median age at transplantation of 13 years, 50% of all current paediatric kidney recipients will need a second graft before the age of 25 years [11].

Overall 5-year transplant survival varies between 44% to 95% [1, 7–9, 12, 14, 18, 21, 23–26, 30] at 5 years, 23%–95% at 10 years [1, 7–9, 12, 21, 23–26, 30], 35% at 15 years [8] and 21–36% at 20 years [1, 6, 8].

Graft survival of repeat transplants has been reported as being equal to or slightly reduced that of first grafts [1, 43]. When DDs were used, graft survival rates at 1, 3 and 5 years were 79%, 69%, and 62%, compared with 74%, 60%, and 47%, respectively, for the repeat transplants; for LDs, they were 91%, 83%, and 76% compared to 86%, 78%, and 72%, respectively, for repeat transplants [43].

#### Effect of recipient age

Age has an important influence on the transplant as well on as patient survival. Until recently, young age was considered to be the most important predictor of outcome: the United Network for Organ Sharing (UNOS) has shown graft survival of 71% and 60% at 1 year and 5 years in those under the age of 2 years at transplantation, 83% and 64% in those aged 3 to 12 years and 85% and 57% in those aged 13 to 21 years [44]. Others have reported similar values for under fives of 67.7% at 1 year, 57.4% at 5 years and 45.2% at 10 years [30]. UK Transplant demonstrated increased risk of early transplant loss, principally due to technical difficulties, in those under 2 years of age, although after this age risk was equal to that in older children [22]. More recent UNOS data has shown that younger age and earlier era of transplantation adversely affect transplant outcome and gave an odds ratio of 2 for increased risk of graft loss in 2 to 5 year olds in comparison with 6 to 12 year olds [42]. However, in the past decade, the effect of young age has become less, such that children under 10 years of age now have the best long-term graft and patient survival rates of all recipients [1, 18]. Causes of graft loss are different in the youngest children, when surgical issues such as venous and arterial thromboses and urological problems predominate. This means that the large proportion of transplant loss is in the first few months after transplantation at this age [20].

Age becomes important again in adolescents, who, as a group, have the worst graft survival rate of all ages [18, 22, 40, 45]. Adolescents have a high percentage of late acute rejection episodes and relatively poor rejection reversal outcomes, compared to the other age groups, suggesting that lack of compliance with immunosuppressive regimens may be an important contributory factor [45]. Non-concordance contributes significantly to transplant loss at this age [22, 46]. However, not only non-concordance may account for the increased graft loss: adolescents have a relatively high incidence of focal segmental glomerulosclerosis (FSGS) (12.3% of causes of ESRF), with decreased graft survival, compared to younger children with FSGS [47].

#### Effect of donor age

Kidneys from deceased donors aged 11–17 years do best, with a 73% 5-year survival rate [18]. Some studies have warned of the increased risk of graft thrombosis in young patients when young donors, particularly under 5 years of age, are used [22, 25], although others have reported equal outcome, with transplant survival of 55% after 5 years, for donors <6 years of age, and of 60% for donors over that age [48], suggesting that the restriction of kidney selection to donors aged >6 years may not be justified. The Italian registry suggests a lower cut-off age of 24 months [31]. More recently, the 5-year graft survival rate from donors <1 year of age has risen to 60% (equal to that of donors aged >50 years of age) and to 70% for donors aged 1 year to 5 years [18]. Therefore, it may be that the use of small kidneys is appropriate in selected patients.

#### Effect of donor type

Living related donation (LRD) has been shown to benefit outcome, with results of 75% and 85% at 10 years, compared to 46% for DDs [12, 49]. Belgian data showed 10% better transplant survival over DD kidneys [21]. Others have shown a benefit of LRD kidneys in the first 5 years after transplantation, although this benefit was lost by 10 years [1]. Possible explanations for this are that non-concordance and the effects of calcineurin inhibitor-induced nephrotoxicity have taken effect by this time and affect recipients of LRD kidneys equally with those receiving DD kidneys. It has been calculated that a 10-year-old child who received a renal transplant in 2000 and is receiving ciclosporin-based immunosuppression can expect a transplant half-life of 13.1 years from an LRD and 10.8 years from a DD [1], although for LD recipients with no acute rejection episodes, half-life has been calculated to be as high as 37.6 years [49].

LRD is of particular benefit to the recipient under 2 years of age. Five-year graft survival for recipients under 2 years old was 86% following LRD and 38% following DD transplantation. Recipients aged between 2 and 18 years over the same time period had a 5-year graft survival rate of 73% following LRD, which was similar to that for recipients less than 2 years of age in this study [20].

#### Effect of pre-emptive transplant

NAPRTCS data show that transplant survival is improved in patients receiving a pre-emptive transplant compared with those undergoing PD and HD. Graft loss resulting from vascular thrombosis is more common in children who undergo PD than in those who on HD [50]. Eurotransplant found a difference in graft survival between those not on dialysis and those on dialysis of 82% and 69%, respectively, at 6 years [40].

It has been postulated that the delaying of transplantation in adolescents may lead to improved adherence, but this is at the cost of longer time on dialysis. The Australia and New Zealand Dialysis and Transplant Registry (ANZDATA) confirmed that 5- and 10-year graft survival rates were significantly worse in adolescents (65% and 50%, respectively) than in recipients aged 2 to 10 years (74% and 58%) and 20 to 29 years (72% and 57%). However, waiting time on dialysis was an independent risk factor for failure of LRD transplants in adolescents, and pre-emptive grafts were associated with a 50% reduction in the risk of graft failure. Delaying transplantation in adolescents may expose them to increased risk of poorer outcomes [51].

#### Recurrent diseases

Diseases that recur after transplantation and, therefore, have a potential to affect outcome include FSGS, membranoproliferative glomerulonephritis (MPGN) and haemolytic uraemic syndrome (HUS) [42, 47, 52, 53]. Oxalate will continue to be deposited in the transplant if liver transplantation is not undertaken in patients with hyperoxaluria. Nephrotic syndrome can recur in patients with congenital nephrotic syndrome, and anti-glomerular basement membrane (anti-GBM) nephritis in patients with Alport’s syndrome, both due to the development of antibody to the ‘missing’ protein. FSGS is the most feared of all, as it recurs in approximately 30% of transplants, conferring a relative risk of transplant loss of 1.27 in comparison to other diseases [42, 47, 54]. Readers are referred to a review of this subject [55].

#### Human leukocyte antigen matching

Donor-specific HLA mismatching is a risk for poor outcome [17]. Worst outcome has been observed for two HLA-DR mismatched grafts, while 000 and favourably matched kidneys (100, 010, 110 HLA-A, -B, -DR mismatches) survived longest [56]. Sensitised patients [panel reactive antibodies (PRA) > 40%] [11] also have a poorer outcome, and donor antigen-specific hyporeactivity correlates with better graft function [57].

#### Immunosuppression

There have been considerable changes in the use of immunosuppression over the years. NAPRTCS has reported that the use of ciclosporin has decreased from 82.3% in 1996 to 20.7% in 2003. In contrast, use of tacrolimus has increased from 5.5% to 67.1% over the same period. There has also been a move away from azathioprine (AZA), from 56.4% to 1.9%, towards mycophenolate mofetil (MMF), use of which is now approximately 57.4%. Antibody induction has also moved away from antithymocyte globulins (ATGs) and OKT3 to anti-IL2 receptor blockers. The use of steroid-sparing regimens is also becoming common [17].

Clearly, changes in immunosuppression to more powerful agents over the years will have affected transplant outcome. There is evidence that the use of MMF in association with ciclosporin is associated with better outcome at 5 years than a similar regimen using AZA, transplant survival’s being 90.7% for MMF and 68.5% for AZA patients. Cumulative rejection-free survival was also better in the MMF group, at 51.2% versus 37.0% in AZA patients [58]. As absence of rejection is associated with better outcome [49], the projected half-life was 14.4/4.5 years in patients with rejection and 18.7/14.5 years without rejection in the MMF/AZA groups, respectively [58].

There is also evidence that tacrolimus is more effective than ciclosporin: a randomised trial of steroids and azathioprine with either tacrolimus or ciclosporin demonstrated that tacrolimus was significantly more effective than ciclosporin in both preventing acute rejection and maintaining graft function, with a 4-year transplant survival rate of 86% and 69%, respectively, and glomerular filtration rates (GFRs) of 71.5 ml/min/1.73 m^2^ and 53.0 ml/min/1.73 m^2^ body surface area, respectively [59].

#### Concordance with therapeutic regimens

Non-concordance with therapeutic regimens is a difficult problem to deal with. It affects patients and families at all ages, but particularly so at adolescence. A study of concordance evaluated by cyclosporine levels, attendance at clinic visits, individual interviews and unexplained late graft dysfunction identified that non-concordance was the main factor in late graft loss, accounting for 71% of cases, and was a particular problem in Afro-American recipients [60].

#### Hypertension

Hypertension is very common after transplantation, and its incidence varies with time, ranging from 46%, 40%, and 66% of children at 1, 5, and 10 years, respectively [9]. Other studies give similar results [8, 33, 61]. The presence of hypertension is a significant and independent predictor of poor long-term transplant function, regardless of the number of rejection episodes or transplant function at 1 year [62].

#### Bladder dynamics

Transplantation into an abnormal urinary tract is associated with a high incidence of urological and infectious complications [63]. However, despite this, several studies have found no effect on patient survival or transplant outcome [63–66]. Based on a review of 25 articles on the subject, it has been suggested that bladder reconstruction should be performed before transplantation when clinically indicated [66], although one study suggests that there is no adverse outcome on long-term allograft survival or function from transplantation into the unaugmented valve bladder [67]. Because of the high urological complication rates, careful surveillance of lower urinary tract function by urodynamic evaluation is essential before transplantation. Reflux does not need to be corrected before transplantation, unless it is causing symptoms or infection [68–70].

#### Laparoscpic donor nephrectomy

Laparoscopic donor nephrectomy is associated with a longer operation time and longer warm ischaemia and cold ischaemia times in LDs than is the open approach [71]. However, the number of hospitalisation days and the use of analgesia in laparoscopy donors are lower, and there are no differences in complications between the techniques. Most importantly, graft outcome does not seem to be affected [72].

### Final height and incidence of obesity

Final height is another factor that is influenced by the era of transplantation: improvements in pre-transplantation management, particularly nutrition, have led to a better height attainment at transplantation, which is recognised as one of the most important factors in final height achievement [73]. Furthermore, the decline in steroid dosing as immunosuppression has also has a positive benefit on growth. Most studies report reduced final height in patients who underwent transplantation in childhood, with up to 44% below the normal range in early reports, improving to 25% more recently [6, 9]. Some studies include patients that have received recombinant human growth hormone (rHGH) and suggest that final height is better than for those not receiving it [74]. Median final heights for women and men, respectively, who did not receive rhGH, were 147.4 and 156.6 cm [75]. They were 151.0 cm [median standard deviation score (SDS) −1.9] and 162.7 cm (median SDS −1.8) in a study containing some patients on rhGH [76], and 156 cm and 165 cm in patients who all received rhGH [77].

However, one study reports good growth in children who did not receive growth hormone: a median height of 158 cm and 166 cm, respectively, for girls and boys older than 17 years, was reduced, in comparison with that of the general population, but it was within the normal range, even though some patients had been treated with the higher doses of steroids used before ciclosporin was available. Many of the patients that had received transplants in the later years of the review period were still only 17 years old, so they may not have attained their final height [1].

Obesity, defined by a body mass index (BMI) >95th centile, is increasing in the transplant population (12.4% after 1995 and 8% before 1995) [36], and seems to be more common in girls [61].

### Psychosocial outcome

In spite of a high re-transplantation rate and the presence of significant morbidity, renal transplantation in children can lead to attainment of a productive and satisfying life, with a high degree of rehabilitation in adulthood. It has to be remembered, however, that most studies are based on returns of questionnaires, which inevitably leads to selection bias. Psychosocial outcome seems to be better after transplantation than for patients on dialysis [78].

#### Employment

Several studies have revealed satisfactory employment levels, reported as follows: 81% employed [1]; 61.5% able to work, 43.4% working [79]; 86% employed [6]; 73% employed versus 72% in the general population, and 6.5% unemployed versus 10.5% in the general population, and 18.7% receiving a disablement pension [75]; 82% employed [33]; and 72% in paid work [80]. The range of occupations is broad, with a normal average working time each week; 91% were satisfied with their ability to perform at work or school, and only 5% were dissatisfied [81].

#### Relationships

Successful relationships are reported in the majority of studies [80, 81] as follows: 50% married, and the overwhelming majority reported satisfaction in their sexual lives [33]; 50% of women and 27% of men married [72]; 67% of women and 25% of men married [1]; and 40% married, 27% had children [80]. The striking observation, however, was the very low incidence of men with children, both overall (7%) and in comparison with the women (24%), both in London [1], in the French study, when 8.3% of men had offspring in comparison to 27% of the women [75]. The cause of this finding is unclear.

In the US study, 89% were satisfied with life in general, 95% said health never or seldom interfered with family life, and 95% felt that health and drug side effects were of no or minor concern in sexual relationships. Only 3% felt that health was a problem in maintaining a sexual relationship (41% were not sexually active). Only 4% stated that health often interfered with their social life; 98% met friends on a regular basis; 76% were satisfied with personal relationships, and 8% were dissatisfied [81].

#### Physical morbidity

In a US study of those who had undergone transplantation between 1967 and 1999, nearly half were severely short and 27% were obese. Rates of hypertension, bone and joint symptoms, fractures, hypercholesterolaemia, and cataracts were high. In spite of significant remaining health issues, 95% reported their health as “fair” or “good”, 61% reported “no” or “minor” physical limitations, and 82% described themselves as “just as” or “more content than others” [33]. Co-morbidity was found in 40% of all patients in the Dutch study: motor, hearing, or visual disabilities were found in 19%. Bone disease, headaches, itching, and tremors were the most reported disabling problems [8]. Other complications included osteoporosis in 53% of patients, avascular bone necrosis in 13%, post-transplantation diabetes mellitus in 10%, and hypertension in 60% [82]. In the study from Norway, minor cataracts without visual disturbance were found in 45% of patients [9]. Sensorineural hearing loss was documented in 18% of patients receiving transplants before the age of 5 years in Finland, and 15% received anti-convulsive treatment after transplantation [83]. In Germany, 27% suffered from additional disabilities [6]. In the USA, health-related absence from work was non-existent for 93%. Health was rated as good to excellent by 91% and fair by 9%. The future was regarded as hopeful or promising by 80% [81].

#### Education

In the French study the distribution of educational level was lower than national averages: 27.4% were at the lowest level versus 3% of the general population, 41.4% were at the middle level, 31.2% had reached the baccalaureate level, and 11% had followed a university course [75]. Compared with that of all age-matched Dutch inhabitants, educational attainment was low [8]. In Finland, 79% of children who had undergone transplantation before the age of 5 years attended ordinary schools and 76% had normal motor performance. The mean intelligence quotient (IQ) was 87, and 6–24% showed impairment in neuropsychological tests [83]. Patients’ scores on achievement tests were influenced by age at diagnosis and by the mother/caregiver’s lower achievement [84].

#### Independence

In the French study, 46% lived in their parents’ home and 54% lived independently. There was a correlation between educational level, paid activity, marital life, and independent housing with final height [75]. Rates of dependency on parents were also high in the Dutch study [8].

## Conclusion

Transplantation is the treatment of choice for children with ESRF. It offers the best chances for growth, development and quality of life, and is associated with a lower mortality risk than is dialysis, particularly that due to CVD. For these reasons, many paediatric nephrologists aim for pre-emptive transplantation for their patients, and more and more families are coming forward as potential LDs for their children.

## Questions (answers appear after references)

**For each question (a) to (e), answer true or false**

1. Factors that positively influence long-term outcome:
  - Analysis of cohorts since the 1980s in comparison to before that
  - Non-Caucasian race
  - Proportion of LDs
  - Co-morbidity
  - A high proportion of adolescents
2. Patient survival:
  - LD is of particular benefit to those under 2 years of age
  - Deaths occur much later after transplantation in those who received transplants before they were 5 years of age than after this age
  - Cause of death is most commonly malignancy
  - The incidence of infections as a cause of death is increasing
  - Is adversely affected by obesity
3. Cardiovascular disease
  - Is the commonest cause of death after transplantation
  - Has a lower incidence in dialysis patients than in post-transplantation patients
  - Is a more common cause of death in Afro-Americans than in the white population
  - Hypertension is uncommon after transplantation
  - Is likely to become more common, given the increasing incidence of obesity after transplantation
4. Transplant survival
  - Is much worse in very young children, according to the latest data
  - Recurrence of FSGS has a worse prognosis in adolescents than in younger children
  - LD transplant survival is superior to DD transplant survival in the first 5 years after transplantation
  - Is superior in pre-emptive transplants in adolescents
  - Is unaffected by HLA matching
5. Psychosocial and other factors
  - There is no evidence for improving height prognosis over the years
  - Laparoscopic donor nephrectomy has a negative effect on graft outcome
  - Bone disease is the commonest cause of post-transplantation morbidity
  - Most studies have not shown an adverse effect of abnormal bladder dynamics on outcome
  - Generally, reports of psychosocial outcomes are disappointing
